# Crystal Structure of the Pleckstrin Homology Domain from the Ceramide Transfer Protein: Implications for Conformational Change upon Ligand Binding

**DOI:** 10.1371/journal.pone.0079590

**Published:** 2013-11-18

**Authors:** Jennifer Prashek, Trung Truong, Xiaolan Yao

**Affiliations:** Division of Molecular Biology and Biochemistry, School of Biological Sciences, University of Missouri Kansas City, Kansas City, Missouri, United States of America; University of Alabama at Birmingham, United States of America

## Abstract

Ceramide transfer protein (CERT) is responsible for the nonvesicular trafficking of ceramide from the endoplasmic reticulum (ER) to the *trans* Golgi network where it is converted to sphingomyelin (SM). The N-terminal pleckstrin homology (PH) domain is required for Golgi targeting of CERT by recognizing the phosphatidylinositol 4-phosphate (PtdIns(4)P) enriched in the Golgi membrane. We report a crystal structure of the CERT PH domain. This structure contains a sulfate that is hydrogen bonded with residues in the canonical ligand-binding pocket of PH domains. Our nuclear magnetic resonance (NMR) chemical shift perturbation (CSP) analyses show sulfate association with CERT PH protein resembles that of PtdIns(4)P, suggesting that the sulfate bound structure likely mimics the holo form of CERT PH protein. Comparison of the sulfate bound structure with the apo form solution structure shows structural rearrangements likely occur upon ligand binding, suggesting conformational flexibility in the ligand-binding pocket. This structural flexibility likely explains CERT PH domain’s low affinity for PtdIns(4)P, a property that is distinct from many other PH domains that bind to their phosphoinositide ligands tightly. This unique structural feature of CERT PH domain is probably tailored towards the transfer activity of CERT protein where it needs to shuttle between ER and Golgi and therefore requires short resident time on ER and Golgi membranes.

## Introduction

Members of the sphingolipid family are important bioactive lipid molecules involved in a wide variety of processes such as cell growth, apoptosis, senescence, migration and inflammation [1]. As a key intermediate in sphingolipid metabolism, ceramide is synthesized in the endoplasmic reticulum (ER) and then transferred to the Golgi apparatus to be further processed into sphingomyelin (SM) and glucosylceramide. While vesicular trafficking is responsible for the pool of ceramide used for glucosylceramide synthesis, the delivery of ceramide from ER to Golgi for SM synthesis is carried out by a cytosolic lipid transfer protein, the ceramide transfer protein (CERT) [2], [3], [4]. Loss of CERT function leads to ceramide accumulation in the ER and impaired SM synthesis [4].

**Figure 1:**
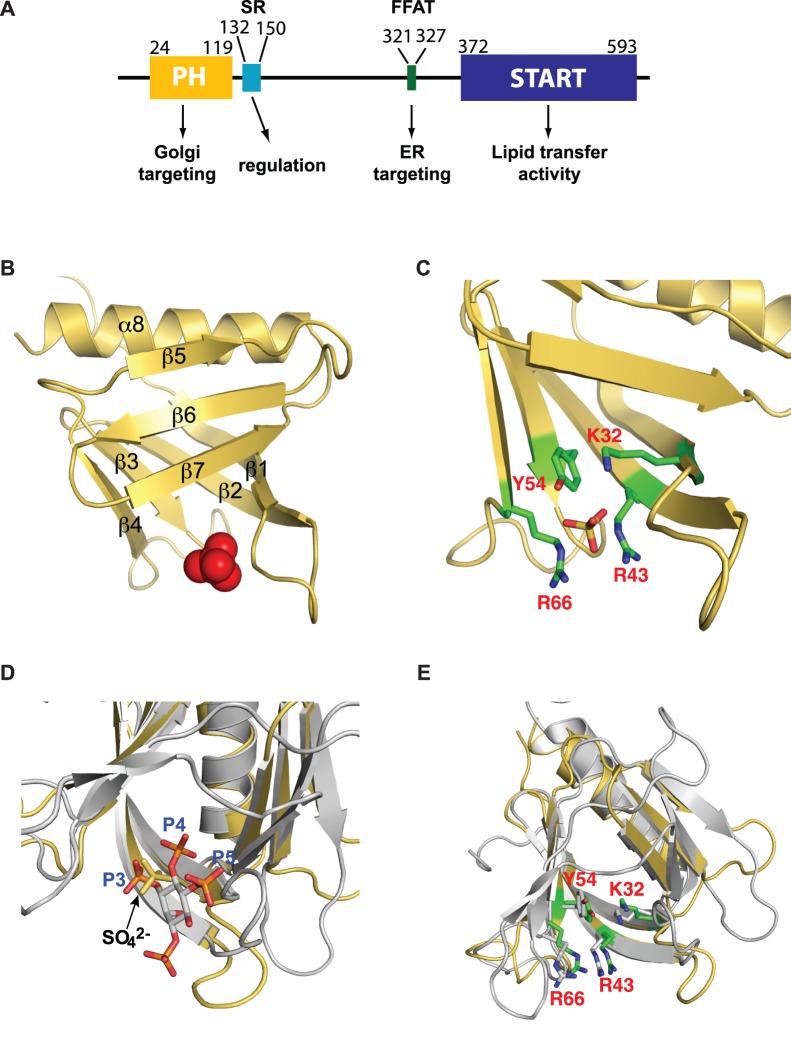
Crystal structure of CERT PH domain with a bound sulfate. (A) Domain structure of CERT. The domain boundaries are determined by SMART [46], [47]. (B) Cartoon representation of CERT PH domain crystal structure with the bound sulfate shown in red spheres. (C) Residues that form hydrogen bonds with the sulfate are labeled and shown in sticks. (D) Overlay of CERT PH structure (pdb code 4HHV, in yellow) and GRP1 PH domain complexed with Ins(1,3,4,5)P_4_ (pdb code 1FGY, in silver). Ins(1,3,4,5)P_4_ and SO_4_ are shown in sticks. (E) Same overlay as in panel (D) but residues that are hydrogen bonded with the sulfate in CERT and corresponding residues in GRP1 are shown in sticks. Residue numbers for CERT are labeled.

CERT is a multidomain protein (Fig. 1A). The N terminal pleckstrin homology (PH) domain is responsible for its localization to the Golgi by binding to phosphatidylinositol-4-phosphate (PtdIns(4)P) that are enriched in the Golgi membrane [4]. Following the PH domain, there is a ∼30-residue stretch rich in serine and threonine residues, thus named serine rich (SR) motif. Phosphorylation of multiple serine and threonine residues in this motif reduces CERT transfer activity and confers functional regulation of the protein [5], [6], [7], [8]. At the C terminus of CERT is a steroidogenic acute regulatory protein (StAR)-related lipid transfer (START) domain that bears the ceramide transfer activity of CERT [4]. Upstream from the START domain, an FFAT (two phenylalanines in an acidic tract) motif interacts with an ER-resident membrane protein, the vesicle associated membrane protein-A (VAP-A), thus targeting CERT to the ER membrane [9]. While the START domain alone bears high ceramide transfer activity *in vitro*, inside the cell, PH domain binding to PtdIns(4)P is essential for CERT function [4]. Importantly, down regulation of CERT activity by phosphorylation is achieved through much reduced binding of PH to PtdIns(4)P [6]. Hence, a detailed understanding of the structural basis of PH binding to PtdIns(4)P-containing membranes is crucial for understanding CERT function and regulation.

PH domains that serve similar function as in CERT are also found in other lipid transfer/binding proteins, including the oxysterol binding proteins (OSBP), the OSBP-related proteins (ORP) and the four-phosphate-adaptor proteins (FAPP) [10], [11], [12]. These PH domains, together with CERT PH, share high sequence identity and functional similarity and constitute a unique group within the PH domain superfamily. Collectively, they are referred to as COF (CERT/OSBP/FAPP) PH domains [13]. Although binding to PtdIns(4)P is required for COF protein localization to the Golgi and is mediated by the PH domain, several studies show COF PH domains have rather modest if any selectivity for PtdIns(4)P against other phosphatidylinositol phosphates (PIP) [10], [11], [13], [14]. It has been shown that FAPP1 and OSBP PH domains also interact with a Golgi localized small GTPase, the ARF1 (ADP-ribosylation factor 1) protein, using a binding interface that is different from PtdIns(4)P association [12], [15]. Therefore, simultaneous binding to PtdIns(4)P and ARF1 ensures specific targeting of FAPP1 and OSBP to the Golgi membrane. To our knowledge, so far there is no direct experimental evidence of CERT protein using the same mechanism. In fact, residues E50 and H70 in FAPP1, which are critical for ARF1 interaction [16], [17], are replaced with valine residues in CERT. These observations suggest the possibility that CERT PH domain does not require ARF1 for Golgi targeting. Rather, either PtdIns(4)P is solely responsible for its Golgi localization or a second binding partner for CERT on the Golgi membrane is yet to be identified.

In this paper, we report a crystal structure of the CERT PH domain and associated biochemical characterization in an effort to understand the structural basis of PH domain mediated CERT localization to the Golgi. The crystal structure contains a bound sulfate anion in the canonical ligand-binding pocket. Nuclear magnetic resonance (NMR) studies show sulfate binding mimics 1,2-dihexanoyl (diC6)-PtdIns(4)P binding to the CERT PH domain, thus the sulfate bound crystal structure likely captures the major features of the PtdIns(4)P bound state. To further investigate the effect of PtdIns(4)P on PH interaction with membrane, we used fluorescence resonance energy transfer (FRET) between Trp residues and 1,6-diphenyl-1,3,5-hexatriene (DPH) embedded in liposomes to measure CERT PH protein affinity for liposomes. Our data show CERT PH domain interaction with lipid vesicles is highly PtdIns(4)P dependent. Moreover, it exhibits more than a thousand fold tighter binding for PtdIns(4)P containing liposomes than for PtdIns(4)P alone in solution. This result is consistent with reported studies on FAPP1 and CERT PH domains where much higher affinities are found for PtdIns(4)P embedded liposomes than for free PtdIns(4)P [13], [16]. A recent study reported the solution NMR structure of the ligand free form CERT PH domain [13]. The same study also showed a basic groove which runs along the middle of the protein is responsible for both specific binding to PtdIns(4)P and nonspecific interactions with liposome head groups. We used the HADDOCK [18] docking program to generate a structural model of diC6-PtdIns(4)P bound to CERT PH protein. The model that is most consistent with the NMR study [13] illustrates that specific PtdIns(4)P binding allows anchoring of PH on the membrane surface in a way that is optimal for nonspecific protein-membrane interactions through basic, aromatic and hydrophobic residues that are conserved within COF PH domains. This study provides structural insight into CERT localization to the Golgi membrane as well as a tool for future investigations of the structural basis of CERT functional regulation.

## Materials and Methods

### Protein Expression and Purification

The CERT PH domain containing residues 20–122 was cloned into the pHis_6_-GB1 plasmid and expressed in *E. coli* strain BL21(DE3). Three extra amino acids (G-E-F) were added before residue 20 as a result of the cloning process. *E. coli* cells were grown in M9 minimal media containing 1 g/L ^15^NH_4_Cl as the sole nitrogen source and 2 g/L glucose (U-^13^C-glucose for uniform ^13^C labeling). Overexpression of recombinant protein was induced by adding 0.5 mM IPTG at ∼0.8 O.D. and the culture was grown for another 12–16 hours at 20°C. Bacteria cells were harvested in 50 mM Tris-HCl pH 8 buffer that also contained 500 mM NaCl and 5 mM β-mercaptoethanol. The protein was first purified with a Ni^2+^-NTA sepharose (QIAGEN) affinity column, followed by an anion exchange Source 15Q (GE healthcare) step. The His_6_-GB1 tag was removed by overnight incubation with Tev protease. The tag and protease were removed by an additional Source 15Q step. Lastly, the protein was exchanged into desired buffer with a Superdex 75 size exclusion column (GE healthcare).

### PH Domain Crystallization, Data Collection, Structure Determination and Refinement

Hampton Research Crystal Screen (HR2-110) was used to initially search for viable crystallization conditions. The initial hit was further optimized and diffraction quality crystals were obtained at 20°C by vapor diffusion of hanging drops over a well solution consisting of 0.1 M sodium citrate pH 6.0, 1.0 M ammonium sulfate. Specifically, 1–2 µL of 10 mg/ml PH protein was mixed with 1 µL of well solution and equilibrated with 400 µL of well solution. Crystals formed within a week. For data collection, crystals were flash frozen in well solution that contained 20% glycerol.

Monochromatic X-ray diffraction data (1.000 Å) were collected at −173°C using beamline 22-BM of the Advanced Photon Source, Argonne National Laboratory. Following data collection, individual reflections were indexed, integrated, and scaled using HKL2000 [19]. Initial phase information was obtained by maximum-likelihood molecular replacement [20], [21]. A search model for molecular replacement was generated by the Phyre2 server [22]. The solution contained two copies of CERT PH protein in the asymmetric unit with an LLG score of 38. Structure refinement was conducted with Refmac with TLS and NCS restrains [21], [23], [24]. One round of individual coordinate and isotropic atomic displacement factors refinement was conducted, and this refined model was used to calculate both 2 mFo-DFc and mFo-DFc difference maps [25]. These maps were used to iteratively improve the model by manual rebuilding in Coot [26], followed by additional refinement of coordinates and atomic displacement factors [21]. Ordered solvent molecules were added during rebuilding in Coot. A final round of modeling and refinement was carried out to 1.75 Å resolution using the native data set described in Table 1.

**Table 1:** Crystallographic data collection and refinement statistics.

Data collection^a^	
Beam line	APS 22-BM
Space group	P2_1_2_1_2_1_
Unit cell dimensions	
a, b, c (Å)	48.13, 54.98, 98.99
α, β, γ (°)	90, 90, 90
Wavelength (Å)	1.000
Resolution (Å)	50.00 (1.75)
Completeness (%)	99.5 (93.9)
Unique reflections	27044
Used reflections	26986
Redundancy (fold)	13.9
<I>/<σI>	13.8
R_merge_ (%)^b^	6.9 (37.8)
**Refinement**	
Number of molecules/a.u.	2
R_work_/R_free_ (%)^c^	17.9/20.4
Number of atoms	
Protein	1683
Solvent	161
Heterogen	35
Ramachandran plot (%)	
Favored	90.6
Allowed	9.4
Outliers	0.0
RMSD	
Bond lengths (Å)	0.016
Bond angles (°)	1.672
Average B-factor (Å^2^)	36.62

a Numbers in parentheses are for the highest-resolution shell.

b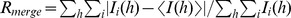 where I_i_(h) is the *i*th measurement of the reflection h, and <I(h)> is a weighted mean of all weighted measurement of h.

c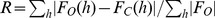 R_work_ and R_free_ were calculated from the working and test reflection sets, respectively. The test set constituted 5% of the total reflections not used in refinement.

The final model contains two PH molecules, which corresponds to a Matthews coefficient of 2.28 Å^3^/Da and a solvent content of 53.8%. Electron density corresponding to residues 20–120 of CERT was modeled for both chains within the asymmetric unit. Additional information and refinement statistics for the structure is presented in Table 1. The coordinates and refined structure factors have been deposited in the RCSB database with the accession code **4HHV**. All CERT PH structure figures were generated by PyMOL [27]. The electrostatic surface of CERT PH domain was calculated with APBS with default settings [28].

### NMR Data Acquisition and Analyses

All NMR experiments were performed at 25°C on a Varian Inova 600 MHz spectrometer. All NMR samples contained 6% D_2_O, 1 mM Tris(2-carboxyethyl)phosphine hydrochloride (TCEP) and a protease inhibitor cocktail. All NMR data were processed using NMRPipe [29] and NMRView [30]. For individual resonance assignments, 800 µM of U-^13^C, ^15^N labeled CERT PH protein in 25 mM Hepes pH 7.5 and 50 mM Na_2_SO_4_ was used [31]. Backbone resonance assignments were carried out with the suite of experiments HNCA, HN(CO)CA, HNCO, HNCACB and CBCA(CO)NH [32]. Titration of diC6-PtdIns(4)P (Cayman Chemicals) ligand into PH protein was performed using 100 µM protein in 25 mM Hepes buffer at pH 7.5 with 100 mM NaCl. A concentrated diC6-PtdIns(4)P stock solution was added to the protein to achieve final ligand concentrations that are at 2x, 3x, 5x, 9x and 11x of the protein concentration. ^15^N-^1^H heteronuclear single quantum correlation (HSQC) spectra were recorded at each ligand concentration and the ^15^N and ^1^H chemical shift changes were used to calculate the normalized chemical shift changes (Δδ) with the following formula:
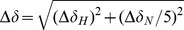
Δδ values at different ligand concentrations were fit to the following equation to obtain K_D_:



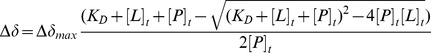
where [L]_t_ is the total ligand concentration; [P]_t_ is the total protein concentration; Δδ is the normalized chemical shift change at a given ligand concentration; Δδ_max_ is the maximal normalized chemical shift change. The software Origin was used for data fitting. Global fit of all affected residues in this manner yields a K_D_ of 470±14 µM. NMR titration study of sulfate ion binding to CERT PH domain was performed similarly to the PtdIns(4)P titration study. Sodium sulfate concentrations that are 10x, 30x, 88x and 200x of PH protein were used. A K_D_ value of 6.8±1.9 mM is obtained from global fitting.

### FRET Measurement of CERT PH Domain Binding to Liposome

FRET between Trp residues of the CERT PH domain and 1,6-diphenyl-1,3,5-hexatriene (DPH) (Invitrogen) embedded in lipid vesicles was used to monitor PH-liposome interaction. 1,2-dioleoyl-*sn*-glycero-3-phosphocholine (DOPC), 1,2-dioleoyl-*sn-*glycero-3-phospho-L-serine (DOPS) and porcine brain PtdIns(4)P were purchased from Avanti lipids. Vesicles containing molar ratios of either DOPC/DOPS/DPH/PI4P (82.8∶9.2∶4:4) or DOPC/DOPS/DPH (86.4∶9.6∶4) were prepared with the extrusion method using 0.2 µm pore size membrane (Avanti lipids). 270 nm was used for fluorescence excitation of Trp residues while DPH emission was monitored at 320–550 nm at different PH protein concentrations. PH proteins were added to 1.4 ml of liposome suspension in a buffer consisting of 25 mM Tris pH 7.5, 100 mM NaCl and 1 mM TCEP. The total vesicle concentration was fixed at 60 µM for all assays. Emission intensity at 485 nm was chosen for FRET analyses to ensure absence of spectral contributions from the protein. The corrected FRET intensity (ΔF) was obtained with the following equation
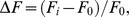
where F_0_ is the intensity in the absence of protein and F_i_ is the intensity at a given PH protein concentration. ΔF was then plotted as a function of protein concentration to obtain the titration curve. By approximation, an apparent K_D_ between protein and liposome was obtained by fitting the titration curve with the following equation [33]:



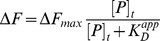
where ΔF_max_ is the maximum corrected FRET intensity and [P]_t_ is the total protein concentration. Average ΔF values from three repetitions were used for data fitting. An apparent K_D_ of 0.34±0.02 µM was obtained between CERT PH and vesicles containing 4% of PtdIns(4)P.

### Molecular Modeling of diC6-PtdIns(4)P - CERT PH Complex Structure

We used the HADDOCK program [18] to generate a structural model of diC6-PtdIns(4)P bound to CERT PH domain. The structural model of diC6-PtdIns(4)P was generated by the PRODRG server [34]. The coordinates of molecule B in the asymmetric unit, with sulfate anion and alternative side chain conformations removed, were used for the calculation. Residues that show significant chemical shift changes (Δδ>one standard deviation) at 11 fold of diC6-PtdIns(4)P were used as experimental restrains for the HADDOCK calculation. The calculation generated five clusters of models. Clusters 1 and 2 have similar HADDOCK and Z scores (HADDOCK score: −85.1±0.9 kcal/mol and Z-Score: −1.2 for cluster 1; HADDOCK score: −81.3±5.9 kcal/mol and Z-Score: −1.0 for cluster 2) while the corresponding scores for the remaining 3 clusters are much worse (HADDOCK scores >−67 kcal/mol and Z-Scores >0.1). Therefore only the four lowest energy structures in clusters 1 and 2 were inspected. In the structure models from cluster 1, PtdIns(4)P binds PH protein with P1 pointing towards helix α8, which is unlikely the case under physiological conditions. Moreover, prior NMR study suggests that the side of the CERT PH protein that encompasses the β1–β2, β5–β6 loops, part of β6, β7 strands and part of α8 helix is involved in membrane interaction [13]. Studies on FAPP1 PH domain interaction with micelles give a similar result [16]. Therefore, we chose a structure model from cluster 2 that is most consistent with these studies.

## Results

### Crystal Structure of CERT PH Domain Reveals a bound Sulfate at the Canonical Binding Site

To understand how the CERT PH domain recognizes PtdIns(4)P, we set out to determine its structure by X-ray crystallography. Crystals of the human CERT PH domain spanning residues 20–122 were obtained using ammonium sulfate as a precipitant. The final structure was refined to 1.75 Å resolution. Data collection and analysis statistics are shown in Table 1. The asymmetric unit contains two copies of CERT PH molecules with nearly identical structures except at the β3–β4 loop (Fig. S1). Unless specified otherwise, the structure of molecule B is used in all Figures. CERT PH has an overall fold that is similar to other PH domains. It contains a curved and twisted β-sandwich that is capped by the C terminal α-helix at one open end (Fig. 1B). Strands 1 to 4 form one side of the β-sandwich while strands 5 to 7 form the opposing β-sheet. Opposite to the capping helix, the other open end of the β-sandwich contains a bound sulfate (Fig. 1B and 1C), which likely originated from the crystallization solution that contained 1.0 M ammonium sulfate. The sulfate forms hydrogen bonds with residues K32, R43, Y54 and R66 (Fig. 1C), all of which are highly conserved among many PH domains and involved in interactions with the PIP ligand [35], [36]. Bound sulfate or phosphate ions have been observed in other PH domain crystal structures where they usually occupy either P3 or P4 position of the intrinsic PIP ligand [35], [37], [38]. Alignment of the CERT PH crystal structure with the Ins(1,3,4,5)P_4_-bound GRP1 (general receptor for phosphoinositides isoform 1) PH domain structure shows the sulfate in CERT PH domain crystal structure is situated close to P3 of the Ins(1,3,4,5)P_4_ molecule in GRP1 structure (Fig. 1D). Moreover, the sulfate-interacting residues K32, R43, Y54 and R66 in CERT and corresponding residues in GRP1, K273, R284, R305 and Y295, adopt very similar orientations (Fig. 1E). In GRP1, these residues are involved in hydrogen bond formations with both P3 and P4 groups (Table 2). This implies the possibility that the P4 in CERT may situate at a position that is similar to either P4 or P3 in GRP1 PH-ligand complex structure.

**Table 2:** Comparison of conserved residues in CERT and GRP1 PH domains involved in hydrogen bond formation with phosphate groups.

CERT PH	GRP1 PH
K32	K273 (P3, P4)
R43	R284 (P3)
R66	R305 (P3)
Y54	Y295 (P4)
none	H355 (P4)

### Sulfate Binding to CERT PH Domain Resembles PtdIns(4)P Binding

**Figure 2:**
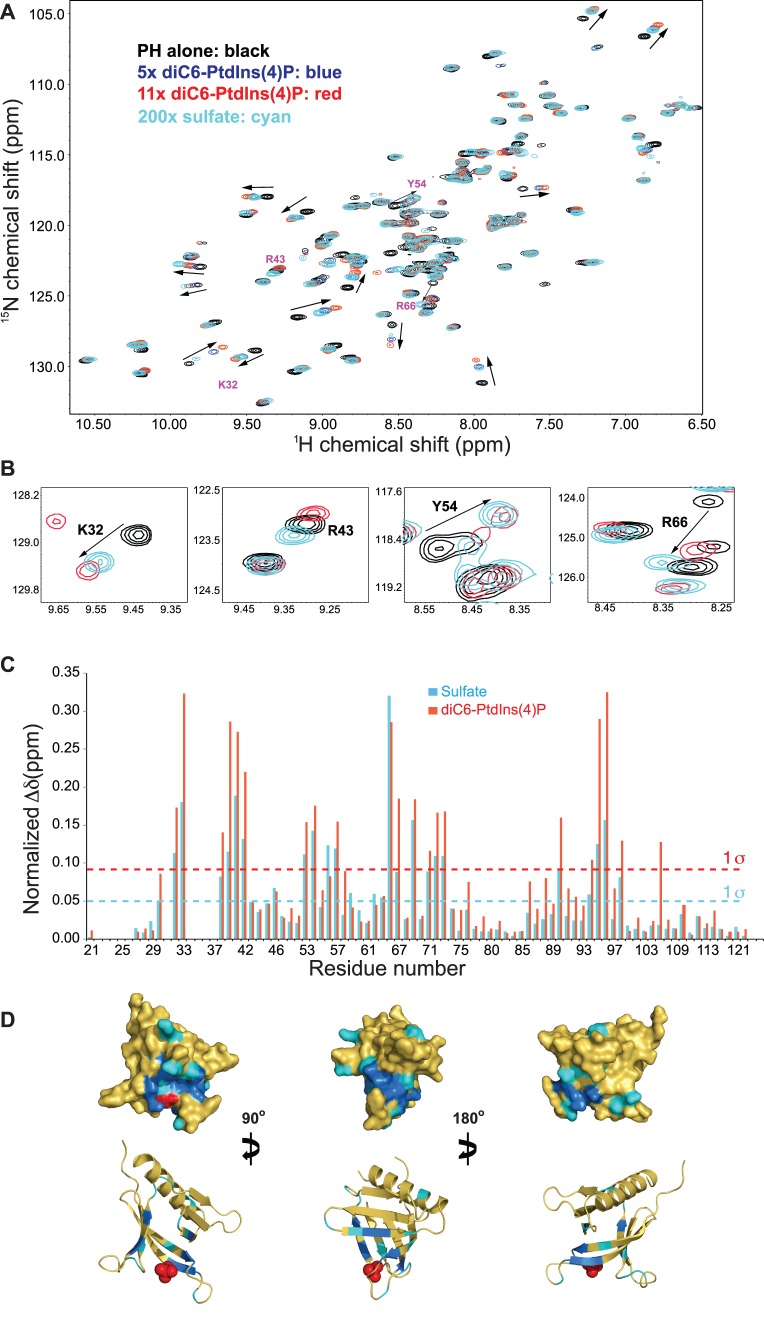
Sulfate binding affects CERT PH domain similarly as diC6-PtdIns(4)P binding. (A) ^15^N-^1^H HSQC spectra overlay of the following: PH alone (black); with 5x (blue) and 11x (red) of diC6-PtdIns(4)P; in the presence of 200x of Na_2_SO_4_ (cyan). (B) ^15^N-^1^H HSQC spectra overlay of the following: PH alone (black); in the presence of 11x of diC6-PtdIns(4)P (red); in the presence of 200x of Na_2_SO_4_ (cyan) for resides K32, R43, Y54 and R66. (C) Plot of Δδ values for all assigned residues at 11x of diC6-PtdIns(4)P (red) and 200x of Na_2_SO_4_ (cyan). The two dotted lines indicate Δδ values that are above 1 standard deviations (1σ) for sodium sulfate (cyan) and diC6-PtdIns(4)P (red) respectively. (D) Residues that have Δδ>2σ (blue) and 1σ<Δδ<2σ (cyan) in the presence of 11x of diC6-PtdIns(4)P are mapped onto the crystal structure.

While our attempts to crystallize the CERT PH protein bound to either Ins(1,4)P_2_ or diC6-PtdIns(4)P were not successful, we suspect that the sulfate anion bound crystal structure may mimic the PtdIns(4)P ligand bound state of the protein. To test this hypothesis, we performed NMR chemical shift perturbation (CSP) analyses to compare the binding of diC6-PtdIns(4)P and sulfate ion to CERT PH domain. The ^15^N-^1^H HSQC spectra of CERT PH protein at different sodium sulfate concentrations are shown in Fig. S2A and S2D. Clearly, addition of sulfate leads to extensive chemical shift perturbations in the protein. The binding between sulfate ion and CERT PH domain manifests as fast exchange, similar to the binding between PtdIns(4)P and CERT PH protein (Figs. 2A and S2B). Importantly, as can be observed from Fig. 2A, residues affected by PtdIns(4)P binding are also perturbed by the presence of sulfate. Moreover, in almost all cases, peaks affected by sulfate binding move in the same direction as those affected by diC6-PtdIns(4)P binding. In particular, residues K32, R43, Y54 and R66, which are responsible for sulfate interaction, have similar chemical shift values at close to saturating concentrations of PtdIns(4)P and sulfate (Fig. 2B), suggesting similar conformations of these residues in the sulfate bound and PtdIns(4)P bound forms. The normalized chemical shift changes (Δδ) across all assigned residues at 11 fold excess of diC6-PtdIns(4)P and 200 fold excess of sodium sulfate are shown in Fig. 2C. Overall, PtdIns(4)P binding leads to larger magnitude of chemical shift changes compared to sulfate binding. Nevertheless, almost all residues affected by diC6-PtdIns(4)P binding also show chemical shift perturbations upon addition of Na_2_SO_4_ (Fig. 2A and 2C). Importantly, a salient feature observed from Fig. 2C is that residues that have large Δδ values (>1σ) in the presence of PtdIns(4)P are also the ones that show significant changes in the presence of sulfate ion. Conversely, residues that are minimally perturbed by PtdIns(4)P binding are essentially unaffected by sulfate ion presence. These observations further support that sulfate binding leads to similar perturbations of CERT PH domain as PtdIns(4)P binding does. Residues that show significant chemical shift changes at 11 fold excess of diC6-PtdIns(4)P are mapped onto CERT PH crystal structure (Fig. 2D, blue: Δδ>2σ; cyan: 1σ<Δδ<2σ). Significant changes are found on β strands 1, 2, 3, 4 and 7, with clustering near the β1–β2 and β3–β4 loop regions. Overall, the region of the protein affected by PtdIns(4)P binding suggests CERT PH domain uses the canonical binding pocket for PtdIns(4)P interaction [39]. It can also be observed from Fig. 2D that the perturbed residues cradle the bound sulfate ion, providing further support that the sulfate in the crystal structure likely captures the major features of PtdIns(4)P binding to CERT PH.

While the NMR CSP studies provide evidence for the similarity between sulfate anion and PtdIns(4)P binding to CERT PH domain, they also indicate the two binding events are not identical. First of all, at near saturating concentrations, diC6-PtdIns(4)P leads to much larger chemical shift changes than sulfate ion does (Fig. 2A, 2B and 2C). Secondly, as can be observed from the representative titration curves of PtdIns(4)P and sulfate binding to CERT PH proteins (Fig. S2C and S2E), sulfate anion exhibits much weaker affinity towards CERT PH protein than PtdIns(4)P does. The K_D_ between diC6-PtdIns(4)P and CERT PH domain is determined to be 470±14 µM while sulfate ion binds about 14 fold weaker with a K_D_ of 6.8±1.9 mM. These differences suggest that the inositol ring, the additional phosphate group, and perhaps even the acyl chains in diC6-PtdIns(4)P also contribute to the binding energy and lead to more pronounced structural changes in CERT PH protein than the sulfate anion.

### CERT PH Domain Binding to Liposome is PtdIns(4)P Dependent

**Figure 3:**
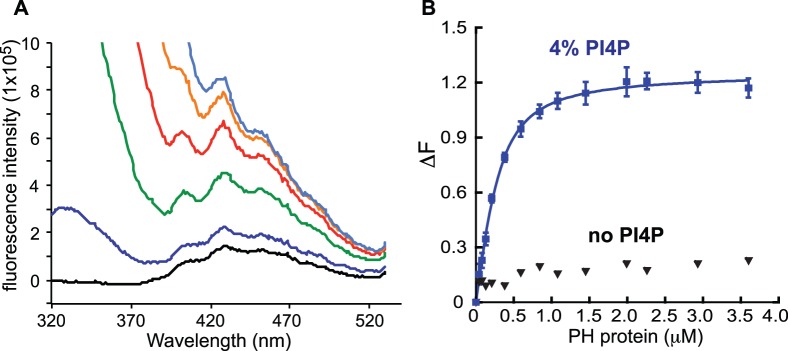
CERT PH binding to membrane measured by FRET. (A) Fluorescence emission spectra of DPH in liposome at different CERT PH protein concentrations (µM): 0 (black), 0.08 (blue), 0.21 (green), 0.38 (red), 1.08 (orange) and 3.6 (light blue). (B) Plot of normalized FRET intensity as a function of protein concentration. Blue circles: liposomes contain 4% of PtdIns(4)P; black triangles: no PtdIns(4)P.

PH domain recognition of PtdIns(4)P is required for CERT localization to the Golgi and disruption of this binding compromises its ceramide transfer activity inside the cell [4]. We measured binding affinity of CERT PH domain to liposomes by FRET between Trp residues of the CERT PH domain and DPH molecules embedded in liposomes. FRET experiments were performed on two types of liposomes: those that contain 4% of PtdIns(4)P and those that do not contain any PtdIns(4)P. For liposomes with PtdIns(4)P, increasing PH protein concentration led to higher FRET intensity between Trp and DPH (Fig. 3A). A plot of corrected FRET intensity versus PH protein concentration is shown in Fig. 3B (blue squares). An apparent K_D_ of 0.34±0.02 µM was obtained from data fitting. In the absence of PtdIns(4)P, addition of PH protein had no effect on DPH emission intensity, i.e., no FRET intensity is observed (Fig. 3B, black triangles). These data show CERT PH domain association with lipid vesicles is directly dependent on PtdIns(4)P.

### CERT PH and diC6-PtdIns(4)P Complex Structure Model Generated by HADDOCK

**Figure 4:**
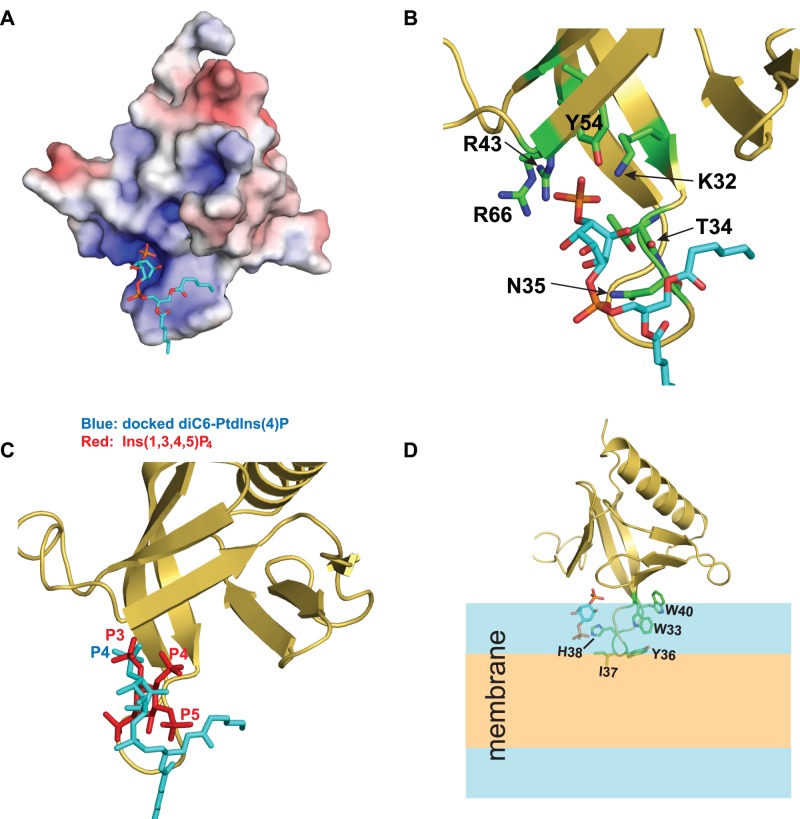
Molecular modeling of CERT PH interaction with PtdIns(4)P. (A) Electrostatic surface of the HADDOCK model with the docked diC6-PtdIns(4)P shown in sticks. (B) Residues that interact with PtdIns(4)P in the structure model are labeled and shown in sticks. (C) Overlay of the HADDOCK model with the ligand bound GRP1 PH domain (1FGY). GRP1 PH ligand Ins(1,3,4,5)P_4_ is shown in red while the diC6-PtdIns(4)P is shown in cyan. (D) A structure model for CERT PH domain associated with PtdIns(4)P containing membrane. The polar region of the membrane is shown in blue and the nonpolar region is shown in orange. For clarity, the acyl chains of diC6-PtdIns(4)P are removed.

To gain further insight into the biophysical basis of CERT targeting to PtdIns(4)P enriched Golgi membranes, we used the HADDOCK [18] software to dock diC6-PtdIns(4)P onto CERT PH domain. Details of the HADDOCK model are shown in Fig. 4. The inositol phosphate moiety of diC6-PtdIns(4)P lies in the highly basic canonical ligand binding pocket of the CERT PH domain (Fig. 4A). Notably, P4 assumes a position that is similar to the sulfate anion in the crystal structure and forms hydrogen bonds with K32, R43, Y54 and R66 (Fig. 4B). In addition, T34 backbone carbonyl and N35 side chain amide are also involved in PtdIns(4)P interactions (Fig. 4B). All these PtdIns(4)P-interacting residues adopt similar conformations as in the crystal structure. Alignment of this structure model with the ligand bound GRP1 PH structure shows P4 in the CERT PH-PtdIns(4)P complex occupies the same position as P3 in the GRP1-Ins(1,3,4,5)P_4_ complex (Fig. 4C). Consequently the bound PtdIns(4)P is roughly parallel to the β1–β2 loop and allows anchoring of CERT PH domain through this loop. A model of membrane associated CERT PH domain based on the HADDOCK modeling is shown in Fig. 4D. The protein docks onto the membrane primarily through the β1–β2 loop. The basic H38 likely engages in electrostatic interactions with membrane head groups while I37, which is located at the tip of the β1–β2 loop, likely is involved in hydrophobic interactions with the lipid acyl chains. In addition, a clustering of aromatic residues W33, Y36 and W40 from this loop may engage in nonspecific protein-membrane interactions as well. These additional protein-membrane interactions likely account for the much higher affinity for the membrane than for the free ligand exhibited by CERT PH domain.

## Discussions

In this study, we present the first crystal structure of CERT PH domain with a sulfate bound at the canonical ligand-binding pocket. Solution NMR studies show that sulfate and PtdIns(4)P binding to CERT PH domain lead to similar perturbations of the protein. This suggests that the sulfate-bound crystal structure likely mimics the PtdIns(4)P associated one. We also show that CERT PH protein interaction with liposome depends directly on PtdIns(4)P. Molecular modeling by HADDOCK provides a plausible model for CERT PH domain binding to PtdIns(4)P containing membranes. The model illustrates CERT PH domain also utilizes basic, aromatic and hydrophobic residues in the β1–β2 loop region to engage in nonspecific interactions with membranes, as suggested by prior studies [13], [16].

The two copies of CERT PH molecules in the asymmetric unit have distinct conformations in the β3–β4 loop. In molecule B, a major portion of this loop points outward and residue R66 from this region is hydrogen bonded with the bound sulfate (Fig. S1B, S1D). In molecule A, the β3–β4 loop points downward and K56 replaces R66 to interact with the sulfate (Fig. S1B, S1C). The rest of sulfate interacting residues, K32, R43 and Y54 share the same conformation between the two molecules (Fig. S1C, S1D). A recent study revealed that mutating K56 to alanine does not affect the affinity between PH protein and PtdIns(4)P containing liposomes. On the other hand, mutation of R66 to alanine reduces the affinity by 13 fold [13]. These two copies of PH molecule possibly reflect conformational dynamics within the protein in solution. Structure B likely represents the major conformation while structure A a minor one. An in-depth understanding of CERT PH protein conformational dynamics and whether the A conformation plays any role in CERT PH domain function would require further experimental investigations. Substantial protein conformational flexibility is further observed from the comparison of CERT PH crystal structure with the Apo form solution structure [13] (Fig. S3C). While the overall fold is similar, local conformational differences are observed between the two. One important difference lies in the highly conserved residue K32. It is situated inside the canonical binding pocket and interacts with the bound sulfate in the crystal structure. In the solution structure, however, K32 side chain resides on the surface of the protein and points away from the binding pocket. Consequently, it needs to undergo conformational changes when binding to PtdIns(4)P. Interestingly, the positive charge feature provided by the K32 side chain in the crystal structure is maintained in the solution structure by flipping R43 upward to occupy the same position (Fig. S3C). It is possible that a basic side chain is required in this location to engage in cation-π interactions [40] with the nearby W95 to maintain protein stability. Sequence alignment of COF PH domains shows the aromatic character of W95 is conserved across all COF PH domains (Fig. S4).

Conformational dynamics can also be inferred from the crystal structure of FAPP1 PH domain where a large part of the β3–β4 loop is not visible (Fig. S3A, S3B). Moreover, residue K41 of FAPP1, which is equivalent to CERT R66 and implicated in PtdIns(4)P binding [16], points away from the PtdIns(4)P binding pocket, similar to the A structure of CERT PH domain but distinct from the B structure (Fig. S3A, S3B). These observations imply the possibility that CERT and FAPP1 PH domains sample similar conformational states. The structural flexibilities observed in both the CERT and FAPP1 PH domains at least partly explain their low affinity and rather modest selectivity towards free PtdIns(4)P in solution [10], [11], [13], [16]. In contrast to the apo form structures of FAPP1 and CERT PH domains, which differ substantially in the ligand binding pocket from the sulfate bound CERT PH crystal structure, in the solution structure of ORP11 PH domain, residues that would contribute to sulfate binding maintain conformations similar to those seen in the CERT PH crystal structure (Fig. S3D), suggesting that ORP11 PH domain might exhibit less structural flexibility and bind to PtdIns(4)P with higher affinity compared to CERT and FAPP1 PH domains.

Our FRET measurement obtained a 0.3 µM K_D_ between CERT PH domain and PtdIns(4)P containing liposomes. This is more than a thousand fold higher than its affinity for free diC6-PtdIns(4)P in solution. Earlier studies also show CERT PH domain exhibits several hundred fold higher affinity towards PtdIns(4)P embedded lipid vesicles than free PtdIns(4)P [13]. Similar observations have also been made in FAPP1 and OSBP PH domains. FAPP1 PH domain binds to diC6-PtdIns(4)P with a K_D_ in the high µM range, but binds to PtdIns(4)P containing liposome with a ∼0.2 µM K_D_ [14], [15], [16]. The K_D_ between OSBP PH domain and PtdIns(4)P containing liposome is 0.7 µM as determined by isothermal titration calorimetry, while no detectible binding is observed towards either the free head group or liposomes that contain phosphatidylinositol [10]. These findings suggest that in addition to interacting with the inositol phosphate head group, nonspecific protein-membrane interactions make a significant energetic contribution to CERT PH domain association with lipid vesicles. These nonspecific interactions likely originate from two sources. First of all, CERT PH domain contains extensive positive charge patches throughout the canonical ligand-binding pocket and the β1–β2 loop (Fig. S5). These regions can engage in electrostatic interactions with the negatively charged head groups on the membrane surface. It has been observed that increasing anionic lipid content in liposomes enhances PH-liposome binding [13]. In addition, aromatic residues such as W33, Y36 and W40 in the β1–β2 loop (Fig. S5) likely interact with the membrane at the membrane-water interfacial region and facilitate anchoring of PH on the membrane surface. Indeed, single point mutations W33A and Y36A lead to 43 and 82-fold decrease in PH-liposome affinity, respectively [13]. Aromatic residues such as Trp and Tyr are known to be enriched in the interfacial region of membrane proteins and contribute to their anchoring in membranes [41], [42], [43]. It is not surprising that they are also critically important in some peripheral membrane protein interaction with membranes. The START domain of CERT interacts with liposome primarily through two adjacent Trp residues [44], [45]. The Y/W feature of Y36 and W40 are conserved in FAPP, OSBP and ORP proteins. W33 is replaced with either a tyrosine or a lysine in some ORP proteins (Fig. S4). These observations imply that usage of aromatic and basic residues in the β1–β2 loop for nonspecific protein-membrane interaction might be a common feature of COF PH domains.

In addition to PtdIns(4)P, CERT PH also binds to lipid vesicles containing other types of PIP molecules, albeit with lower affinity: about twenty fold lower for PtdIns(3)P or PtdIns(5)P and about five fold lower for PtdIns(4,5)P_2_ [13]. FAPP1 and OSBP PH domains only have about two fold higher affinity for PtdIns(4)P than for PtdIns(4,5)P_2_ [14]. In fact, the FAPP1 PH domain binds to free PtdIns(4,5)P_2_ even slightly better than to PtdIns(4)P in solution [16]. As discussed earlier, nonspecific protein-membrane interactions have significant contributions to COF family PH domain binding to PIP containing membranes. Consequently, the nonspecific protein-membrane interactions can influence the selectivity of COF PH domains towards different PIP lipid molecules. A structural model of the CERT PH domain complexed with diC6-PtdIns(4)P illustrates that the head group of diC6-PtdIns(4)P binds to PH with an angle that is roughly parallel to the β1–β2 loop, thus permitting residues from the β1–β2 loop and β7 strand to interact with the membrane (Fig. 4). On the other hand, either PtdIns(3)P or PtdIns(5)P head group association would lead to different orientations of PH protein on the membrane surface and potentially reduces nonspecific protein-membrane interactions. This structural model provides a plausible explanation for the modest selectivity of COF PH domains towards PtdIns(4)P. Interestingly, in a study where the liposomes contained only the neutral phosphatidylcholine and nonspecific protein-membrane electrostatic interaction is minimal, PH domains from two yeast OSBP proteins, Osh1p and Osh2p, bear no selectivity towards PtdIns(4)P against either PtdIns(3,5)P_2_ or PtdIns(4,5)P_2_ [11]. Although this data seems to suggest that COF PH domain selectivity towards PtdIns(4)P has a large dependence on the lipid composition of the membrane, a clear understanding of this needs further detailed and systematic experimental investigation that is currently ongoing. We also note that the structural model presented in Fig. 4 provides only a possible mode of CERT PH docking at the Golgi membrane surface. A detailed understanding of PH orientation and insertion depth when it interacts with the PtdIns(4)P containing membrane would require more experimental investigations.

## Supporting Information

Figure S1 **The two CERT PH molecules in the asymmetrical unit have different conformations in the β3–β4 loop.** (A) Overlay of the two structures. (B) Molecule A (cyan) uses K56 while molecule B (yellow) uses R66 to form hydrogen bond with the sulfate. (C) Sulfate-interacting residues in molecule A. (D) Sulfate-interacting residues in molecule B.
(PDF)

Figure S2 **Sulfate ion binds to CERT PH domain with much weaker affinity than PtdIns(4)P.** (A) ^15^N-^1^H HSQC spectra of CERT PH domain at different sulfate concentrations. (B) A region of ^15^N-^1^H HSQC spectra of CERT PH domain at different diC6-PtdIns(4)P concentrations. (C) Representative titration curves obtained by plotting normalized chemical shift changes (Δδ) as a function of PtdIns(4)P concentration. (D) A region of ^15^N-^1^H HSQC spectra of CERT PH domain at different sodium sulfate concentrations. (E) Representative titration curves of sulfate ion binding to CERT PH protein.
(PDF)

Figure S3 **Comparisons of CERT PH crystal structure with other COF PH structures.** (A) Overlay of FAPP1 PH domain crystal structure (3RCP, cyan) with CERT PH molecule B (yellow). Residues that are hydrogen bonded with sulfate in CERT and the corresponding ones in FAPP1 are shown in sticks. (B) Overlay of FAPP1 PH domain structure (cyan) with CERT PH molecule A (yellow). R66 in CERT and K41 in FAPP1 are shown in sticks. (C) Overlay of CERT PH molecule B (yellow) with NMR solution structure (2RSG, cyan). The red arrows indicate conformational changes from solution structure to crystal structure. (D) Overlay of ORP11 PH domain solution structure (2D9X, cyan) with CERT PH crystal structure (yellow). Residues that are hydrogen bonded with sulfate in CERT and the corresponding ones in ORP11 are shown in sticks.
(PDF)

Figure S4 **Sequence alignment of COF PH domains. Residue numbers of CERT PH domain are labeled.** The alignment is generated by CLUSTALW (48) and displayed with ESpript (49).
(PDF)

Figure S5 **Electrostatic surface of CERT PH domain between ± 5 kT, calculated with APBS, the corresponding cartoon representation of the structure is also shown.** Aromatics residues from β1–β2 loop that likely contribute to nonspecific protein-liposome interaction are shown in sticks.
(PDF)

## References

[1] Hannun YA, Obeid LM (2008) Principles of bioactive lipid signalling: lessons from sphingolipids. Nature reviews Molecular cell biology 9: 139–150. 18216770 10.1038/nrm2329

[2] Bartke N, Hannun YA (2009) Bioactive sphingolipids: metabolism and function. Journal of lipid research 50 Suppl: S91–96 10.1194/jlr.R800080-JLR200 PMC2674734 19017611

[3] Yamaji T, Kumagai K, Tomishige N, Hanada K (2008) Two sphingolipid transfer proteins, CERT and FAPP2: their roles in sphingolipid metabolism. IUBMB life 60: 511–518. 18459163 10.1002/iub.83

[4] Hanada K, Kumagai K, Yasuda S, Miura Y, Kawano M, et al (2003) Molecular machinery for non-vesicular trafficking of ceramide. Nature 426: 803–809. 14685229 10.1038/nature02188

[5] Fugmann T, Hausser A, Schoffler P, Schmid S, Pfizenmaier K, et al (2007) Regulation of secretory transport by protein kinase D-mediated phosphorylation of the ceramide transfer protein. The Journal of cell biology 178: 15–22. 17591919 10.1083/jcb.200612017 PMC2064413

[6] Kumagai K, Kawano M, Shinkai-Ouchi F, Nishijima M, Hanada K (2007) Interorganelle trafficking of ceramide is regulated by phosphorylation-dependent cooperativity between the PH and START domains of CERT. J Biol Chem 282: 17758–17766. 17442665 10.1074/jbc.M702291200

[7] Saito S, Matsui H, Kawano M, Kumagai K, Tomishige N, et al (2008) Protein phosphatase 2Cepsilon is an endoplasmic reticulum integral membrane protein that dephosphorylates the ceramide transport protein CERT to enhance its association with organelle membranes. J Biol Chem 283: 6584–6593. 18165232 10.1074/jbc.M707691200

[8] Tomishige N, Kumagai K, Kusuda J, Nishijima M, Hanada K (2009) Casein kinase I{gamma}2 down-regulates trafficking of ceramide in the synthesis of sphingomyelin. Molecular biology of the cell 20: 348–357. 19005213 10.1091/mbc.E08-07-0669 PMC2613112

[9] Kawano M, Kumagai K, Nishijima M, Hanada K (2006) Efficient trafficking of ceramide from the endoplasmic reticulum to the Golgi apparatus requires a VAMP-associated protein-interacting FFAT motif of CERT. J Biol Chem 281: 30279–30288. 16895911 10.1074/jbc.M605032200

[10] Levine TP, Munro S (1998) The pleckstrin homology domain of oxysterol-binding protein recognises a determinant specific to Golgi membranes. Current biology: CB 8: 729–739. 9651677 10.1016/s0960-9822(98)70296-9

[11] Yu JW, Mendrola JM, Audhya A, Singh S, Keleti D, et al (2004) Genome-wide analysis of membrane targeting by S. cerevisiae pleckstrin homology domains. Molecular cell 13: 677–688. 15023338 10.1016/s1097-2765(04)00083-8

[12] Levine TP, Munro S (2002) Targeting of Golgi-specific pleckstrin homology domains involves both PtdIns 4-kinase-dependent and -independent components. Current biology: CB 12: 695–704. 12007412 10.1016/s0960-9822(02)00779-0

[13] Sugiki T, Takeuchi K, Yamaji T, Takano T, Tokunaga Y, et al (2012) Structural basis for the Golgi association by the pleckstrin homology domain of the ceramide trafficking protein (CERT). The Journal of biological chemistry 287: 33706–33718. 22869376 10.1074/jbc.M112.367730 PMC3460467

[14] Stahelin RV, Karathanassis D, Murray D, Williams RL, Cho W (2007) Structural and membrane binding analysis of the Phox homology domain of Bem1p: basis of phosphatidylinositol 4-phosphate specificity. The Journal of biological chemistry 282: 25737–25747. 17581820 10.1074/jbc.M702861200

[15] He J, Scott JL, Heroux A, Roy S, Lenoir M, et al (2011) Molecular basis of phosphatidylinositol 4-phosphate and ARF1 GTPase recognition by the FAPP1 pleckstrin homology (PH) domain. The Journal of biological chemistry 286: 18650–18657. 21454700 10.1074/jbc.M111.233015 PMC3099681

[16] Lenoir M, Coskun U, Grzybek M, Cao X, Buschhorn SB, et al (2010) Structural basis of wedging the Golgi membrane by FAPP pleckstrin homology domains. EMBO reports 11: 279–284. 20300118 10.1038/embor.2010.28 PMC2854595

[17] Roy A, Levine TP (2004) Multiple pools of phosphatidylinositol 4-phosphate detected using the pleckstrin homology domain of Osh2p. The Journal of biological chemistry 279: 44683–44689. 15271978 10.1074/jbc.M401583200

[18] de Vries SJ, van Dijk M, Bonvin AM (2010) The HADDOCK web server for data-driven biomolecular docking. Nature protocols 5: 883–897. 20431534 10.1038/nprot.2010.32

[19] Otwinowski Z, Minor W (1997) Processing of X-ray Diffraction Data Collected in Oscillation Mode. Methods in Enzymology 276: 307–326. 10.1016/S0076-6879(97)76066-X 27754618

[20] McCoy AJ, Grosse-Kunstleve RW, Adams PD, Winn MD, Storoni LC, et al (2007) Phaser crystallographic software. Journal of applied crystallography 40: 658–674. 19461840 10.1107/S0021889807021206 PMC2483472

[21] Afonine PV, Grosse-Kunstleve RW, Echols N, Headd JJ, Moriarty NW, et al (2012) Towards automated crystallographic structure refinement with phenix.refine. Acta crystallographica Section D, Biological crystallography 68: 352–367. 22505256 10.1107/S0907444912001308 PMC3322595

[22] Kelley LA, Sternberg MJ (2009) Protein structure prediction on the Web: a case study using the Phyre server. Nature protocols 4: 363–371. 19247286 10.1038/nprot.2009.2

[23] Murshudov GN, Vagin AA, Dodson EJ (1997) Refinement of macromolecular structures by the maximum-likelihood method. Acta crystallographica Section D, Biological crystallography 53: 240–255. 15299926 10.1107/S0907444996012255

[24] Winn MD, Ballard CC, Cowtan KD, Dodson EJ, Emsley P, et al (2011) Overview of the CCP4 suite and current developments. Acta crystallographica Section D, Biological crystallography 67: 235–242. 21460441 10.1107/S0907444910045749 PMC3069738

[25] Adams PD, Afonine PV, Bunkoczi G, Chen VB, Davis IW, et al (2010) PHENIX: a comprehensive Python-based system for macromolecular structure solution. Acta crystallographica Section D, Biological crystallography 66: 213–221. 20124702 10.1107/S0907444909052925 PMC2815670

[26] Emsley P, Lohkamp B, Scott WG, Cowtan K (2010) Features and development of Coot. Acta crystallographica Section D, Biological crystallography 66: 486–501. 20383002 10.1107/S0907444910007493 PMC2852313

[27] Delano WL (2002) The PyMOL Molecular Graphic System. http://wwwpymolorg.

[28] Baker NA, Sept D, Joseph S, Holst MJ, McCammon JA (2001) Electrostatics of nanosystems: application to microtubules and the ribosome. Proceedings of the National Academy of Sciences of the United States of America 98: 10037–10041. 11517324 10.1073/pnas.181342398 PMC56910

[29] Delaglio F, Grzesiek S, Vuister GW, Zhu G, Pfeifer J, et al (1995) NMRPipe: a multidimensional spectral processing system based on UNIX pipes. Journal of biomolecular NMR 6: 277–293. 8520220 10.1007/BF00197809

[30] Johnson BA, Blevins RA (1994) NMR View: A computer program for the visualization and analysis of NMR data. Journal of biomolecular NMR 4: 603–614. 22911360 10.1007/BF00404272

[31] Sugiki T, Yoshiura C, Kofuku Y, Ueda T, Shimada I, et al (2009) High-throughput screening of optimal solution conditions for structural biological studies by fluorescence correlation spectroscopy. Protein science: a publication of the Protein Society 18: 1115–1120. 19388076 10.1002/pro.92 PMC2771313

[32] Cavanagh J, Fairbrother WJ, Palmer AGI, Skelton NJ, Rance M (2006) Protein NMR Spectroscopy: Principles and Practice: Elsevier.

[33] Corbin JA, Dirkx RA, Falke JJ (2004) GRP1 pleckstrin homology domain: activation parameters and novel search mechanism for rare target lipid. Biochemistry 43: 16161–16173. 15610010 10.1021/bi049017a PMC3625374

[34] Schuttelkopf AW, van Aalten DM (2004) PRODRG: a tool for high-throughput crystallography of protein-ligand complexes. Acta crystallographica Section D, Biological crystallography 60: 1355–1363. 15272157 10.1107/S0907444904011679

[35] Lemmon MA, Ferguson KM (2001) Molecular determinants in pleckstrin homology domains that allow specific recognition of phosphoinositides. Biochemical Society transactions 29: 377–384. 11497993 10.1042/bst0290377

[36] DiNitto JP, Lambright DG (2006) Membrane and juxtamembrane targeting by PH and PTB domains. Biochimica et biophysica acta 1761: 850–867. 16807090 10.1016/j.bbalip.2006.04.008

[37] Ferguson KM, Kavran JM, Sankaran VG, Fournier E, Isakoff SJ, et al (2000) Structural basis for discrimination of 3-phosphoinositides by pleckstrin homology domains. Molecular cell 6: 373–384. 10983984 10.1016/s1097-2765(00)00037-x

[38] Lietzke SE, Bose S, Cronin T, Klarlund J, Chawla A, et al (2000) Structural basis of 3-phosphoinositide recognition by pleckstrin homology domains. Molecular cell 6: 385–394. 10983985 10.1016/s1097-2765(00)00038-1

[39] Lemmon MA (2007) Pleckstrin homology (PH) domains and phosphoinositides. Biochemical Society symposium: 81–93. 10.1042/BSS0740081 PMC3777418 17233582

[40] Gallivan JP, Dougherty DA (1999) Cation-pi interactions in structural biology. Proceedings of the National Academy of Sciences of the United States of America 96: 9459–9464. 10449714 10.1073/pnas.96.17.9459 PMC22230

[41] Killian JA, von Heijne G (2000) How proteins adapt to a membrane-water interface. Trends in biochemical sciences 25: 429–434. 10973056 10.1016/s0968-0004(00)01626-1

[42] Wimley WC, White SH (1996) Experimentally determined hydrophobicity scale for proteins at membrane interfaces. Nature structural biology 3: 842–848. 8836100 10.1038/nsb1096-842

[43] Yau WM, Wimley WC, Gawrisch K, White SH (1998) The preference of tryptophan for membrane interfaces. Biochemistry 37: 14713–14718. 9778346 10.1021/bi980809c

[44] Kudo N, Kumagai K, Matsubara R, Kobayashi S, Hanada K, et al (2010) Crystal structures of the CERT START domain with inhibitors provide insights into the mechanism of ceramide transfer. Journal of molecular biology 396: 245–251. 20036255 10.1016/j.jmb.2009.12.029

[45] Kudo N, Kumagai K, Tomishige N, Yamaji T, Wakatsuki S, et al (2008) Structural basis for specific lipid recognition by CERT responsible for nonvesicular trafficking of ceramide. Proc Natl Acad Sci U S A 105: 488–493. 18184806 10.1073/pnas.0709191105 PMC2206563

[46] Schultz J, Milpetz F, Bork P, Ponting CP (1998) SMART, a simple modular architecture research tool: identification of signaling domains. Proceedings of the National Academy of Sciences of the United States of America 95: 5857–5864. 9600884 10.1073/pnas.95.11.5857 PMC34487

[47] Letunic I, Doerks T, Bork P (2012) SMART 7: recent updates to the protein domain annotation resource. Nucleic acids research 40: D302–305. 22053084 10.1093/nar/gkr931 PMC3245027

