# Supplementary figures and images for: Crystal Structure of the Pleckstrin Homology Domain from the Ceramide Transfer Protein: Implications for Conformational Change upon Ligand Binding
Source: PLoS One. 2013 Nov 18;8(11):e79590. doi: 10.1371/journal.pone.0079590 (PMC3832616; doi:10.1371/journal.pone.0079590)

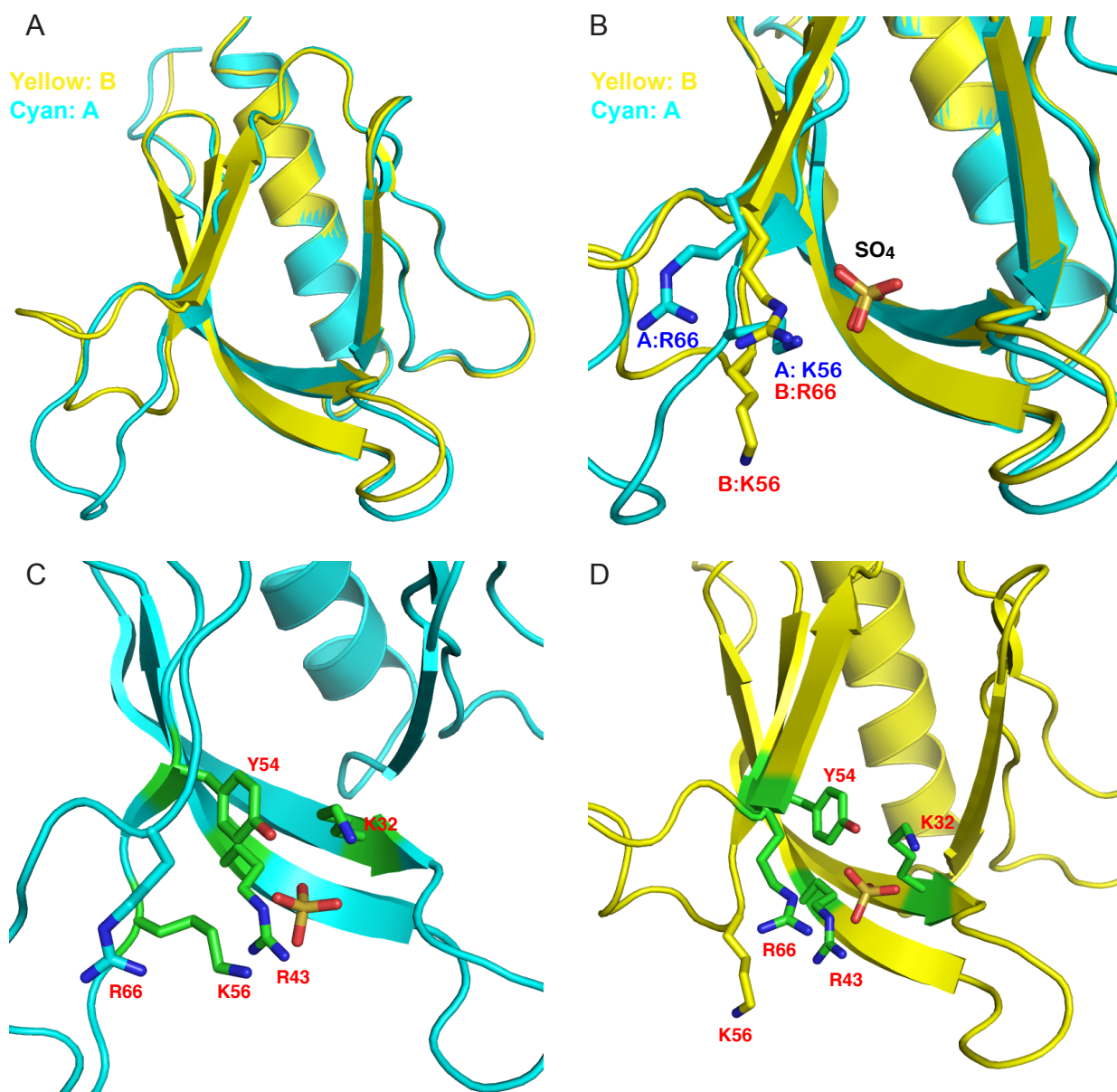

Figure S1

Supplement: Figure S1 — The two CERT PH molecules in the asymmetrical unit have different conformations in the β3–β4 loop. (A) Overlay of the two structures. (B) Molecule A (cyan) uses K56 while molecule B (yellow) uses R66 to form hydrogen bond with the sulfate. (C) Sulfate-interacting residues in molecule A. (D) Sulfate-interacting residues in molecule B. (PDF) [file pone.0079590.s001.pdf]

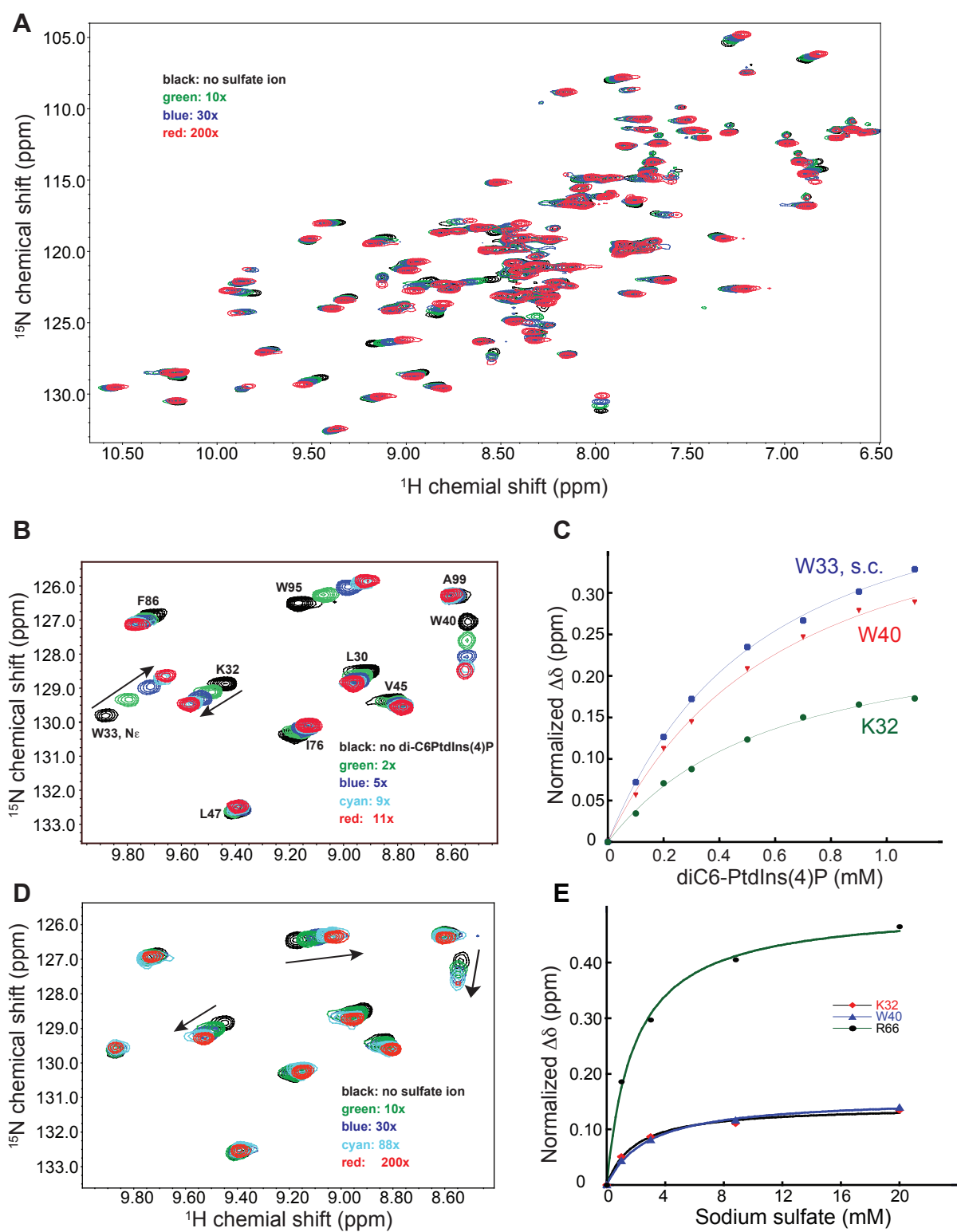

Figure S2

Supplement: Figure S2 — Sulfate ion binds to CERT PH domain with much weaker affinity than PtdIns(4)P. (A) 15N-1H HSQC spectra of CERT PH domain at different sulfate concentrations. (B) A region of 15N-1H HSQC spectra of CERT PH domain at different diC6-PtdIns(4)P concentrations. (C) Representative titration curves obtained by plotting normalized chemical shift changes (Δδ) as a function of PtdIns(4)P concentration. (D) A region of 15N-1H HSQC spectra of CERT PH domain at different sodium sulfate concentrations. (E) Representative titration curves of sulfate ion binding to CERT PH protein. (PDF) [file pone.0079590.s002.pdf]

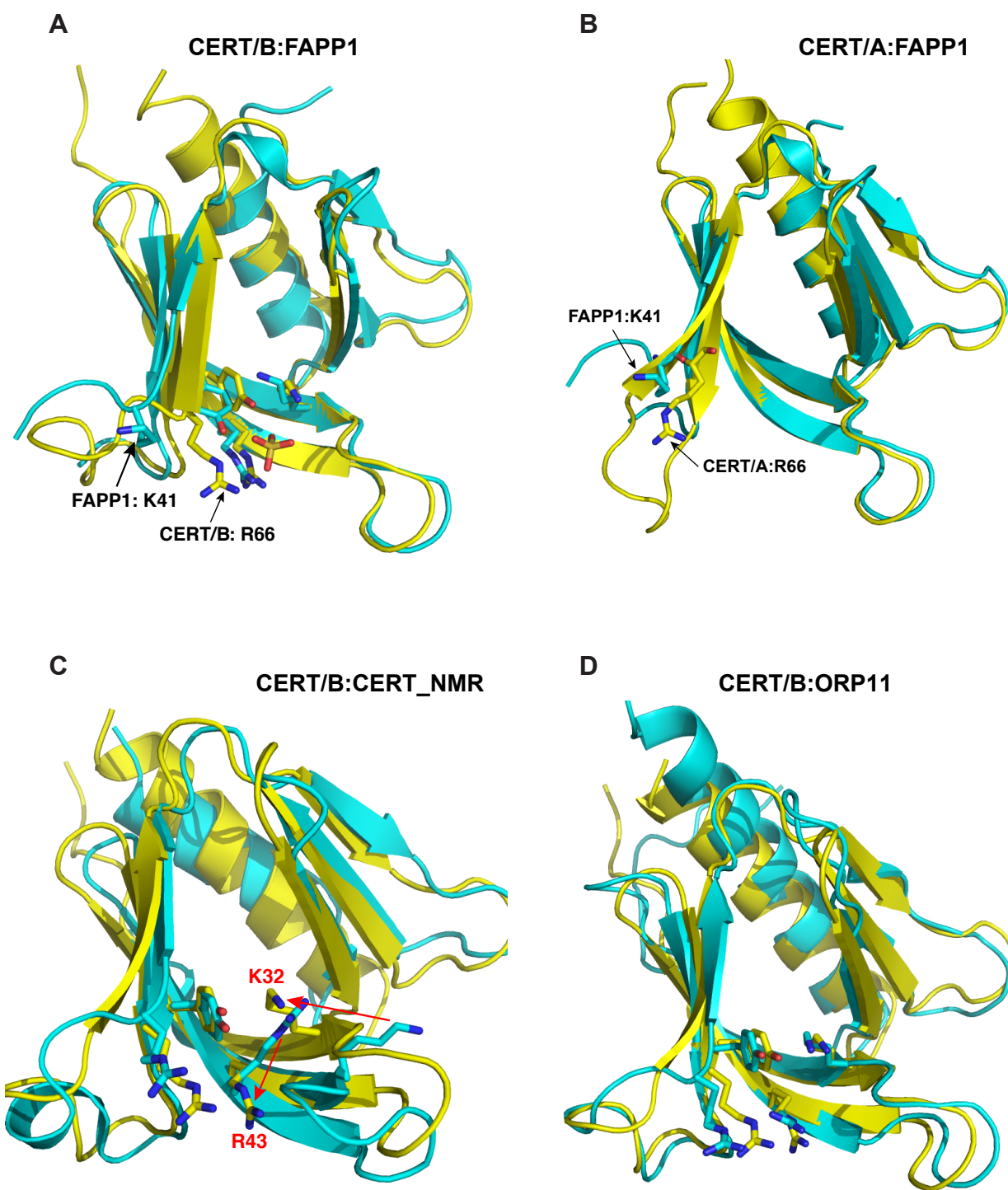

Figure S3

Supplement: Figure S3 — Comparisons of CERT PH crystal structure with other COF PH structures. (A) Overlay of FAPP1 PH domain crystal structure (3RCP, cyan) with CERT PH molecule B (yellow). Residues that are hydrogen bonded with sulfate in CERT and the corresponding ones in FAPP1 are shown in sticks. (B) Overlay of FAPP1 PH domain structure (cyan) with CERT PH molecule A (yellow). R66 in CERT and K41 in FAPP1 are shown in sticks. (C) Overlay of CERT PH molecule B (yellow) with NMR solution structure (2RSG, cyan). The red arrows indicate conformational changes from solution structure to crystal structure. (D) Overlay of ORP11 PH domain solution structure (2D9X, cyan) with CERT PH crystal structure (yellow). Residues that are hydrogen bonded with sulfate in CERT and the corresponding ones in ORP11 are shown in sticks. (PDF) [file pone.0079590.s003.pdf]

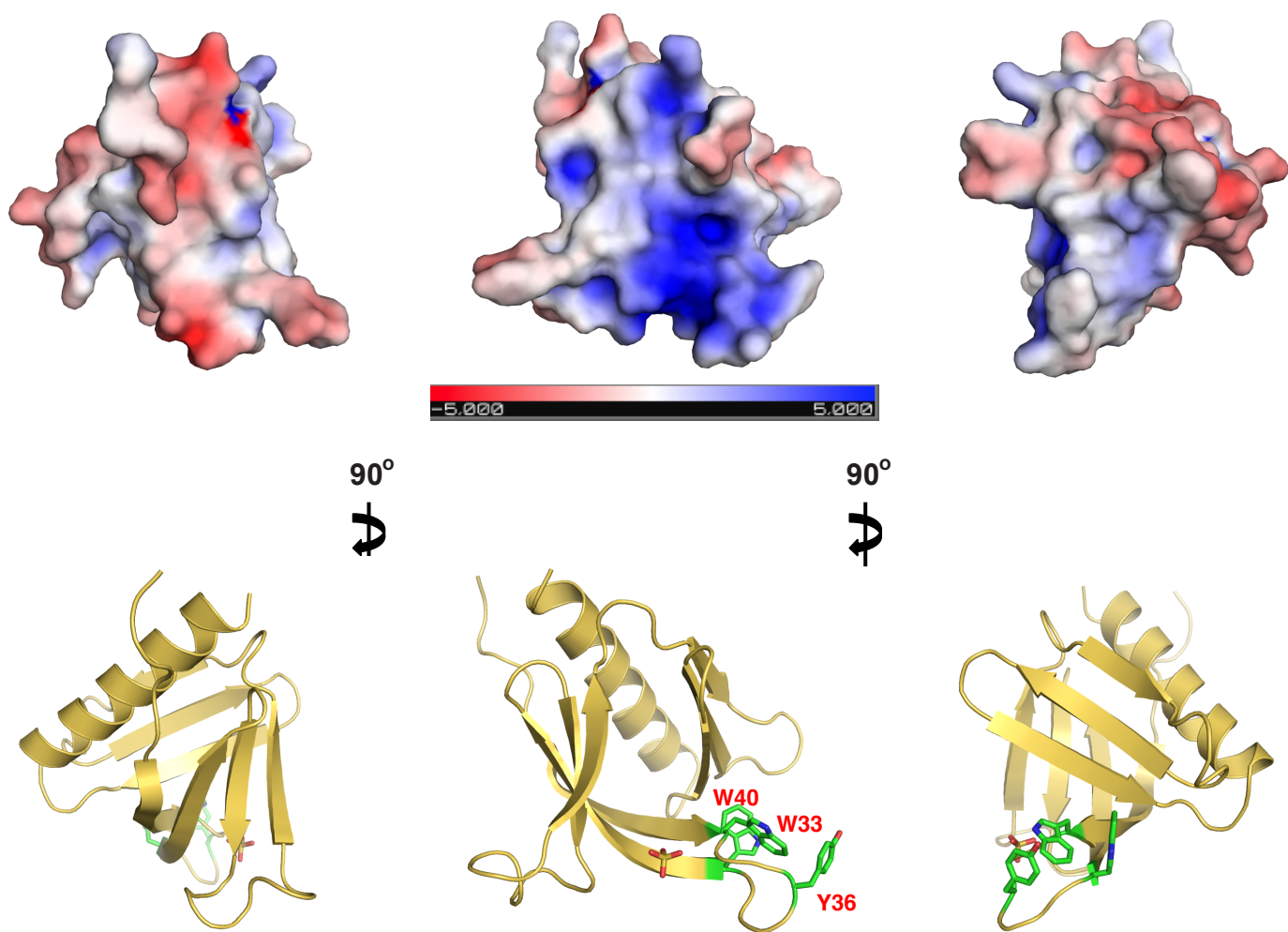

Figure S5

Supplement: Figure S5 — Electrostatic surface of CERT PH domain between ± 5 kT, calculated with APBS, the corresponding cartoon representation of the structure is also shown. Aromatics residues from β1–β2 loop that likely contribute to nonspecific protein-liposome interaction are shown in sticks. (PDF) [file pone.0079590.s005.pdf]
