# Supplementary material for: Crystal Structure of the Pleckstrin Homology Domain from the Ceramide Transfer Protein: Implications for Conformational Change upon Ligand Binding
Source: PLoS One. 2013 Nov 18;8(11):e79590. doi: 10.1371/journal.pone.0079590 (PMC3832616; doi:10.1371/journal.pone.0079590)
Supplement: Figure S4 — Sequence alignment of COF PH domains. Residue numbers of CERT PH domain are labeled. The alignment is generated by CLUSTALW (48) and displayed with ESpript (49). (PDF) [file pone.0079590.s004.pdf]

```
                20        30        40        50
hCERT   ...GPPVERCGVLSKWTNY.IHGWQDRWVVLKN..NALSY
hFAPP1  GPLGSPEFMEGVLYKWTNY.LTGWQPRWFVLDN..GILSY
hFAPP2  ........MEGVLYKWTNY.LSGWQPRWFLLCG..GILSY
hOSBP1  ......SAREGWLFKWTNY.IKGYQRRWFVLSN..GLLSY
hORP3   ......PVQKGFLLKKRKWPLKGWHKRFFYLDK..GILKY
hORP4L  ......DSFEGWLLKWTNY.LKGYQRRWFVLGN..GLLSY
hORP6   ......DKHEGFMLKKRKWPLKGWHKRFFVLDN..GMLKY
hORP7   ......ERQEGHLLKKRKWPLKGWHKRYFVLED..GILHY
hORP9   ......SIMEGPLSKWTNV.MKGWQYRWFVLDYNAGLLSY
hORP10  ......PALEGVLSKYTNL.LQGWQNRYFVLDFEAGILQY
hORP11  ......ENVYGYLMKYTNL.VTGWQYRFFVLNNEAGLLEY

             60        70        80        90
hCERT   YKSEDETEYGCR.GSICLSKAVITPHDFDECRFDISVN.D
hFAPP1  YDSQDDVCKGSK.GSIKMAVCEIKVHSADNTRMELIIPGE
hFAPP2  YDSPEDAWKGCK.GSIQMAVCEIQVHSVDNTRMDLIIPGE
hOSBP1  YRSKAEMRHTCR.GTINLATANITVE..DSCNFIISNGGA
hORP3   AKSQTDIEREKLHGCIDVGLSVMSVKK.SSKCIDLDTE.E
hORP4L  YRNQGEMAHTCR.GTINLSTAHIDTE..DSCGILLTSG.A
hORP6   SKAPLDIQKGKVHGSIDVGLSVMSIKK.KARRIDLDTE.E
hORP7   ATTRQDITKGKLHGSIDVRLSVMSINK.KAQRIDLDTE.D
hORP9   YTSKDKMMRGSRRGCVRLRGAVIGIDDEDDSTFTITVD.Q
hORP10  FVNEQSKHQKPR.GVLSLSGAIVSLSDEAPHMLVVYSANG
hORP11  FVNEQSRNQKPR.GTLQLAGAVISPSDEDSHTFTVNAASG

             100       110       120
hCERT   SVWYLRAQDPDHRQQWIDAIEQHKTESG....
hFAPP1  QHFYMKAVNAAERQRWLVALGSSKASLTDTRT
hFAPP2  QYFYLKARSVAERQRWLVALGSAKACL.....
hOSBP1  QTYHLKASSEVERQRWVTALELAKAKA.....
hORP3   HIYHLKVKSEEVFDEWVSKLRHHRMYR.....
hORP4L  RSYHLKASSEVDRQQWITALELAKAKA.....
hORP6   HIYHLKVKSQDWFDAWVSKLRHHRLYR.....
hORP7   NIYHLKIKSQDLFQSWVAQLRAHRLAH.....
hORP9   KTFHFQARDADEREKWIHALEETILRH.....
hORP10  EMFKLRAADAKEKQFWVTQLRACAKYH.....
hORP11  EQYKLRATDAKERQHWVSRLQICTQHH.....
```

Figure S4
